# Distinct Mesenchymal Alterations in N-Cadherin and E-Cadherin Positive Primary Renal Epithelial Cells

**DOI:** 10.1371/journal.pone.0043584

**Published:** 2012-08-17

**Authors:** Christof Keller, Sven Kroening, Jonathan Zuehlke, Frank Kunath, Bettina Krueger, Margarete Goppelt-Struebe

**Affiliations:** 1 Department of Nephrology and Hypertension, University of Erlangen-Nuremberg, Erlangen, Germany; 2 Department of Urology, University of Erlangen-Nuremberg, Erlangen, Germany; 3 Department of Cellular and Molecular Physiology, University of Erlangen-Nuremberg, Erlangen, Germany; INSERM, France

## Abstract

**Background:**

Renal tubular epithelial cells of proximal and distal origin differ markedly in their physiological functions. Therefore, we hypothesized that they also differ in their capacity to undergo epithelial to mesenchymal alterations.

**Results:**

We used cultures of freshly isolated primary human tubular cells. To distinguish cells of different tubular origin we took advantage of the fact that human proximal epithelial cells uniquely express N-cadherin instead of E-cadherin as major cell-cell adhesion molecule. To provoke mesenchymal alteration we treated these cocultures with TGF-β for up to 6 days. Within this time period, the morphology of distal tubular cells was barely altered. In contrast to tubular cell lines, E-cadherin was not down-regulated by TGF-β, even though TGF-β signal transduction was initiated as demonstrated by nuclear localization of Smad2/3. Analysis of transcription factors and miRNAs possibly involved in E-cadherin regulation revealed high levels of miRNAs of the miR200-family, which may contribute to the stability of E-cadherin expression in human distal tubular epithelial cells. By contrast, proximal tubular epithelial cells altered their phenotype when treated with TGF-β. They became elongated and formed three-dimensional structures. Rho-kinases were identified as modulators of TGF-β-induced morphological alterations. Non-specific inhibition of Rho-kinases resulted in stabilization of the epithelial phenotype, while partial effects were observed upon downregulation of Rho-kinase isoforms ROCK1 and ROCK2. The distinct reactivity of proximal and distal cells was retained when the cells were cultured as polarized cells.

**Conclusions:**

Interference with Rho-kinase signaling provides a target to counteract TGF-β-mediated mesenchymal alterations of epithelial cells, particularly in proximal tubular epithelial cells. Furthermore, primary distal tubular cells differed from cell lines by their high phenotypic stability which included constant expression of E-cadherin. Our cell culture system of primary epithelial cells is thus suitable to understand and modulate cellular remodeling processes of distinct tubular cells relevant for human renal disease.

## Introduction

Epithelial cells possess the unique property to reversibly alter their phenotype and to adopt features of mesenchymal cells. During this process cells lose polarity, E-cadherin-mediated adherens junctions are dissolved, and the cells express mesenchymal markers [1]. This type of plasticity of epithelial cells is well characterized in tumor progression and may contribute to metastasis and invasiveness [2]. In the kidney, tubular epithelial cells exhibit different phenotypes according to their physiological role in each part of the nephron. For example, proximal tubular cells depict a cubical epithelium with an apical brush boarder in accordance with their role in reabsorption of the bigger part of water and solutes whereas distal tubular cells lack a brush boarder and line the tubules as cobble-stone like monolayer. Most notably, proximal tubular cells are the only epithelial cells in the human adult organism which express N-cadherin instead of E-cadherin as major cell-cell adhesion protein [3], [4]. This difference is rarely acknowledged in studies investigating molecular properties of proximal epithelial cells, which may be due to the fact that cell lines derived from human proximal tubules such as HK-2 or HKC-8 express both, N-cadherin and varying levels of E-cadherin. Furthermore, there seems to be species specificity with N-cadherin being expressed in human and rat proximal tubules [5], whereas E-cadherin and N-cadherin are detected in mouse proximal tubules forming distinct complexes with catenins [6].

Phenotypically altered tubular epithelial cells have been observed in chronic kidney disease and it has been proposed that these cells have undergone epithelial-to mesenchymal transition to contribute to interstitial fibrosis. While this concept is under debate [7], [8], dedifferentiation of tubular cells is a common feature of tubular damage during chronic kidney disease and is mostly reversible with proliferating and migrating epithelial cells contributing to repair processes [9].

So far, cell lines of different rodent or human tubular epithelial cells have been established to understand the molecular mechanisms of phenotypic changes of renal tubular cells observed *in vivo* [10], [11]. Interestingly, little is known about the cellular behavior of primary human tubular epithelial cells, which seem to differ from cell lines in terms of mesenchymal transition, e.g. in the ability to express α-smooth muscle actin [3], [12], [13]. Transforming growth factor−β (TGF-β) is considered a major factor driving mesenchymal alterations [9], [14]. By binding to specific receptors, TGF-β activates Smads and alternative signaling pathways thereby modulating the expression of target genes which regulate complex cellular function such as cell growth, apoptosis, differentiation and synthesis of extracellular matrix [15], [16], [17] Primary human cell cultures isolated from human kidney sections consist of proximal and distal tubular epithelial cells [3], and preparations of either type of cells have not been rigorously tested in terms of mesenchymal reactions to TGF-β.

Mesenchymal alterations include rearrangement of the cytoskeleton, which is mediated by small GTPases of the Rho family. While activation of Smad transcriptions factors is the classical signaling pathway of TGF-β, activation of RhoA or RhoC has been reported in various cell types among them epithelial cells. [18], [19], [20], [21]. Downstream of RhoA and RhoC, Rho kinases ROCK1 and ROCK2 are important mediators of alterations of the cytoskeleton and subsequent modulation of gene expression [22]. Inhibition of Rho kinases showed renoprotective effects in various models of chronic kidney injury [23], [24]. Rho kinase isoforms share many biological activities, but may also exert differential functions which seem to be cell type-specific [25]. While targeted deletion of ROCK1 in mice was protective in the heart, no such effects were observed in the obstructed kidney [26], [27], [28]. Due to the limited number of studies, genotype dependent effects cannot be ruled out to contribute to the observed differences. Isoform-specific effects of Rho kinases have been reported in different cells in vitro, e.g. keratinocytes [29], vascular smooth muscle cells [30] or fibroblasts [31], but thus far no data are available in epithelial cells.

Based on clinical studies proximal and distal tubular cells were expected to differ with respect to mesenchymal plasticity [32]. Therefore, we used freshly isolated human cells from healthy parts of tumor nephrectomies to analyze effects of TGF-β on both cell types under polarized and non-polarized cell culture conditions. We show that E-cadherin expressing distal tubular cells largely preserved their phenotype. We identify differences between primary cells and cell lines in expression and regulation of miRNAs of the miR200 family providing a molecular explanation for the stability of E-cadherin. By contrast, N-cadherin expressing proximal tubular cells adopted a mesenchymal phenotype upon treatment with TGF-β. We show that these morphological alterations can be reduced by inhibitors of Rho-kinases, suggesting that these drugs may be useful to stabilize the epithelial phenotype.

## Results

### Proximal Tubular Cells Show a Higher Morphological Plasticity than Distal Tubular Cells

**Figure 1:**
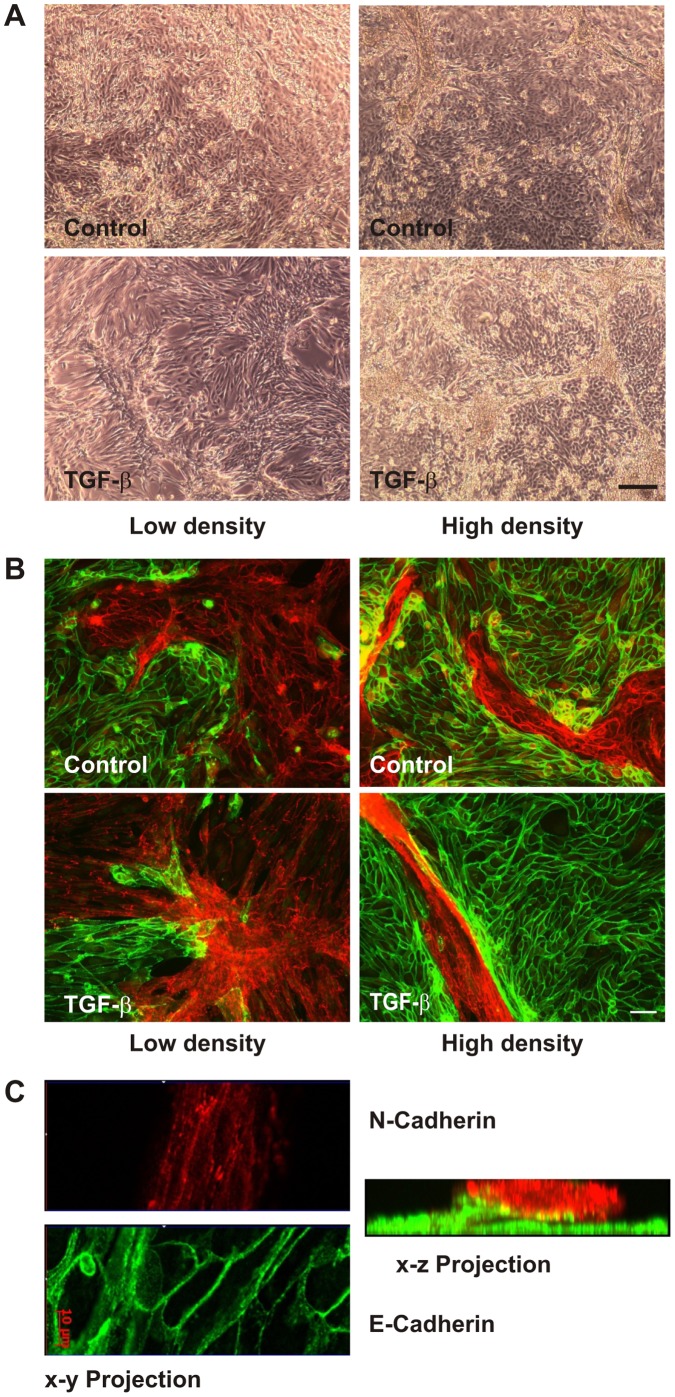
Differential morphological plasticity of proximal and distal hPTECs upon treatment with TGF-β. (A) hPTECs were seeded at passage 1 at low (25 000 cells/cm^2^) or high density (75 000 cells/cm^2^) at day 1. Cells were treated with TGF-β (2 ng/ml) at day 2. Phase contrast images were taken at day 5. Scale bar: 200 µm. (B) Cells were treated as in A. N-Cadherin-positive proximal tubular cells (red) and E-cadherin-positive cells (green) were detected by immunocytochemistry. Scale bar: 50 µm. (C) Cells depicted in Fig. 1B (high density, TGF-β) were analyzed by epifluorescence with ApoTome technique to present x–y and x–z projections, respectively.

Human primary tubular cells (hPTECs) at passage one were seeded on collagen-IV-coated glass cover slips and were then cultivated until day 5 in the absence of serum. At confluence two populations of adherent cells were visible by phase contrast microscopy (Fig. 1A). One population formed a cobble stone-like pattern whereas the other formed three dimensional structures most obviously when the cells were seeded at a higher density (Fig. 1A, control cells).

hPTECs reorganized into patches of cells with homotypic N-cadherin or E-cadherin cell-cell adhesions (Fig. 1B). Distal cells expressing E-cadherin formed a regular pattern and remained adherent even at high densities. N-cadherin positive cells deriving from proximal tubules formed a more irregular pattern and built three-dimensional structures at higher densities. These morphological differences between cells of different origin became even more pronounced upon stimulation with TGF-β (Fig. 1 A/B). Distal tubular cells reacted to TGF-β in a cell density-dependent manner: only low density cells became elongated spindle-like cells, whereas dense cells retained the regular pattern. Even prolonged incubation with TGF-β for up to 6 days did not alter the phenotype of distal cells stably expressing E-cadherin (data not shown). Proximal tubular epithelial cells elongated and formed complex structures. Three-dimensional visualization revealed clusters of N-cadherin-positive cells on top of E-cadherin positive distal cells (Fig. 1C).

Differences in cellular plasticity between proximal and distal tubular cells were confirmed in more than 10 isolations from different donors, and were observed in cells cultured on collagen-IV-or fibronectin-coated glass plates, and on uncoated plastic surfaces.

### Distal Polarized Tubular Cells Retain their Morphology in the Presence of TGF-β

**Figure 2:**
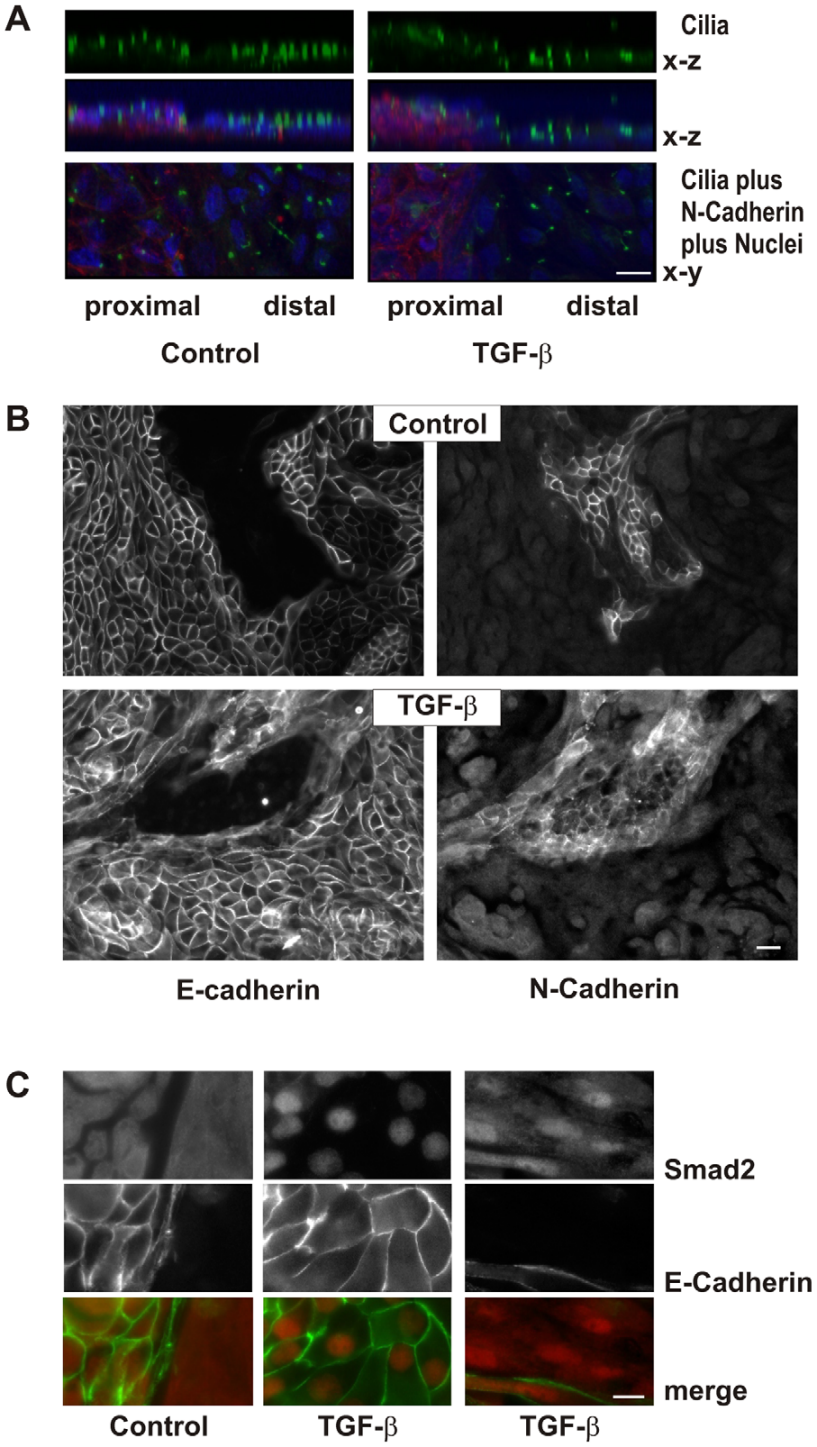
Polarization of primary epithelial cells. (A) hPTECs were cultured in permeable filter inserts for 8 days and then further incubated with TGF-β for 7 days. Cilia were detected by staining with antibodies against acetylated tubulin. Merged images show an overlay of N-cadherin (red), DAPI (blue) and acetylated tubulin (green). X–y and x–z orientation is presented as indicated. Scale bar: 10 µm. (B) hPTECs were treated as described in A. Proximal and distal cells were distinguished by staining for N-cadherin and E-cadherin respectively. Scale bar: 20 µm. (C) Polarized hPTECs were treated with TGF-β for 2 h. Cells were stained with antibodies against E-cadherin and Smad 2/3. Cells not stained with E-cadherin were considered to be proximal hPTECs (right panel). Scale bar: 10 µm.

*In vivo*, epithelial cells show a polarized morphology with the apical side facing the lumen of the tubule and the basolateral side being aligned with the blood vessels. To induce polarization we established a cell culture protocol for freshly isolated tubular cells. 8 days after seeding in transwell inserts, the cells formed a polarized monolayer verified by the apical orientation of cilia (Fig. 2A). Distal cells formed a cobble stone like pattern and surrounded areas of tightly packed proximal cells (Fig. 2B). Polarized cells were additionally characterized by specific staining of distal E-cadherin positive cells with peanut agglutinin and proximal N-cadherin positive cells with antibodies against aminopeptidase N (CD13) (Figure S1). Further incubation of these polarized cells in transwell inserts for 3 to 7 days in the presence or absence of TGF-β did not further alter cell morphology of distal epithelial cells nor did the presence of TGF-β reduce E-cadherin expression (Fig. 2B). Distal cells with established cell-cell contacts were thus rather resistant to morphological alterations induced by TGF-β. By contrast, proximal cells showed a higher flexibility and proved to be less adherent (Fig. 2A/B).

The different responsiveness to TGF-β was not due to a loss of signal transduction in polarized distal cells. In control cells, Smad2/3 immunoreactivity was evenly distributed in the cytosol of the cells, also covering the nucleus (Fig. 2C, left panel). Activation of the cells with TGF-β for 2 h led to Smad2/3 accumulation in the nucleus. In E-cadherin positive distal cells, Smad2/3 staining was confined to the nuclei, whereas in proximal cells, Smad2/3 staining was detectable in the cytosol but enriched in the nucleus (Fig. 2C, right hand panels).

### Molecular Mechanisms Involved in E-cadherin Stability in Distal Tubular Cells

**Figure 3:**
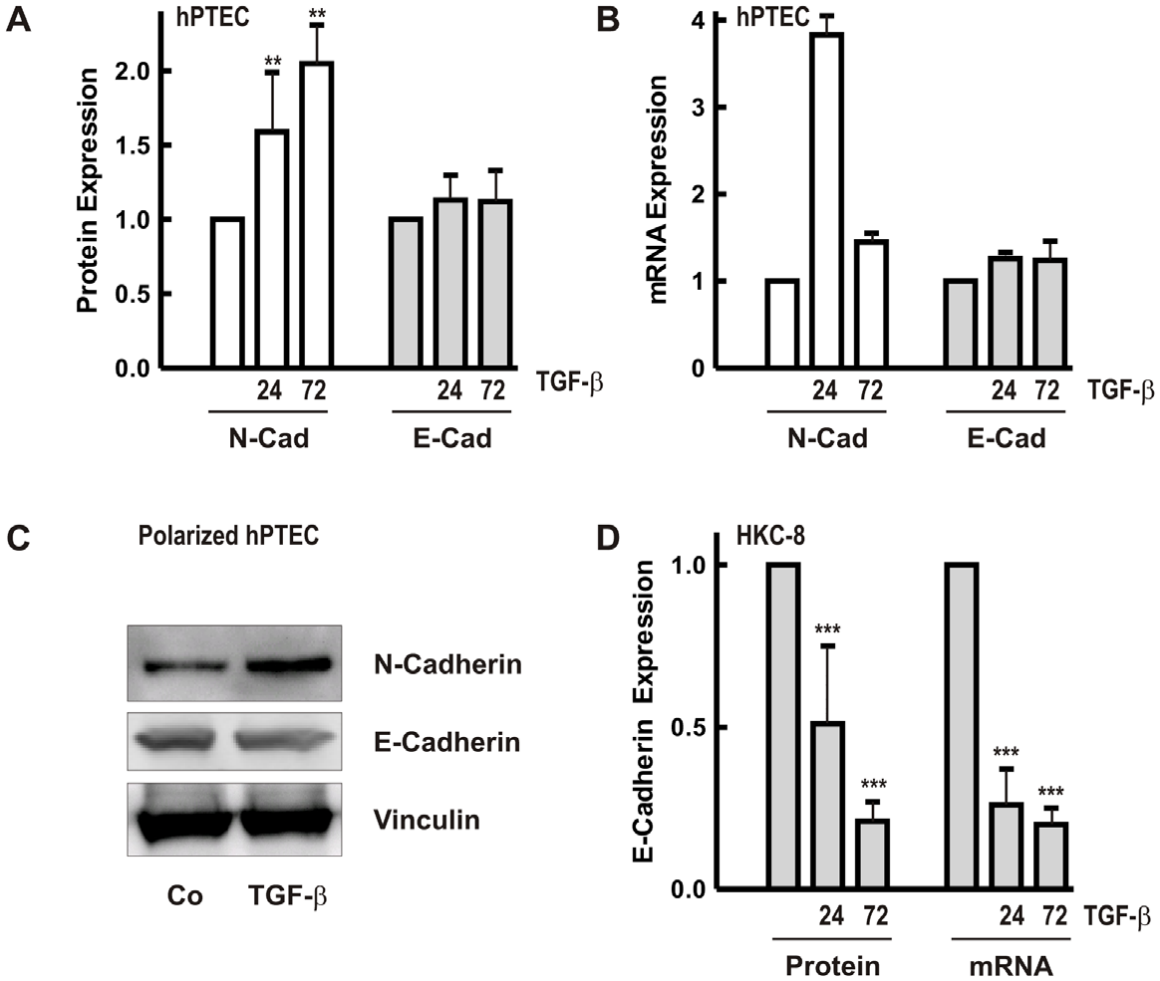
Regulation of N-and E-cadherin expression by TGF-β. (A) hPTECs cultured in plastic dishes were treated with TGF-β (2 ng/ml) for 24 and 72 h. N-and E-cadherin protein expression was detected in cellular homogenates by Western blot analysis. Expression of the respective protein in control cells after 24 or 72 h was set to 1. Data are means +/− SD of 3–5 independent preparations. **p<0.01, one way ANOVA with Dunnett’s post hoc test. (B) Cells were treated as in A. mRNA expression of N-and E-cadherin was determined by quantitative RT-PCR. Expression of N-cadherin or E-cadherin in control cells was set to 1 in each experiment. Data are means of 2–4 independent preparations. (C) Polarized hPTECs were treated with TGF-β for 72 h. E-cadherin and N-cadherin were detected in cellular homogenates by Western blotting. Vinculin served as control. (D) HKC-8 cells were treated with TGF-β for 24 and 72 h. To summarize E-cadherin protein and mRNA of different experiments, expression of the respective control cells was set to 1. Data are means of 4–8 experiments. ***p<0.001, one way ANOVA with Dunnett’s post hoc test.

Immunocytochemistry did not indicate downregulation of E-cadherin in distal hPTECs as reported in other epithelial cells treated with TGF-β. Quantification of E-cadherin protein and mRNA revealed stable expression over 72 h of stimulation with TGF-β (2 ng/ml) with a slight increase detected in some preparations (Fig. 3A/B). This increase may reflect variations in the ratio of proximal and distal cell numbers. By contrast, N-cadherin was consistently upregulated. Results were confirmed in polarized hPTECs with upregulation of N-cadherin and no change in E-cadherin (Fig. 3C).

For comparison, we also analyzed the proximal tubular cell line HKC-8, which expresses N-cadherin as dominant cell-cell adhesion molecule, but also E-cadherin. Upon stimulation with TGF-β E-cadherin protein and mRNA were rapidly downregulated (Fig. 3D).

**Figure 4:**
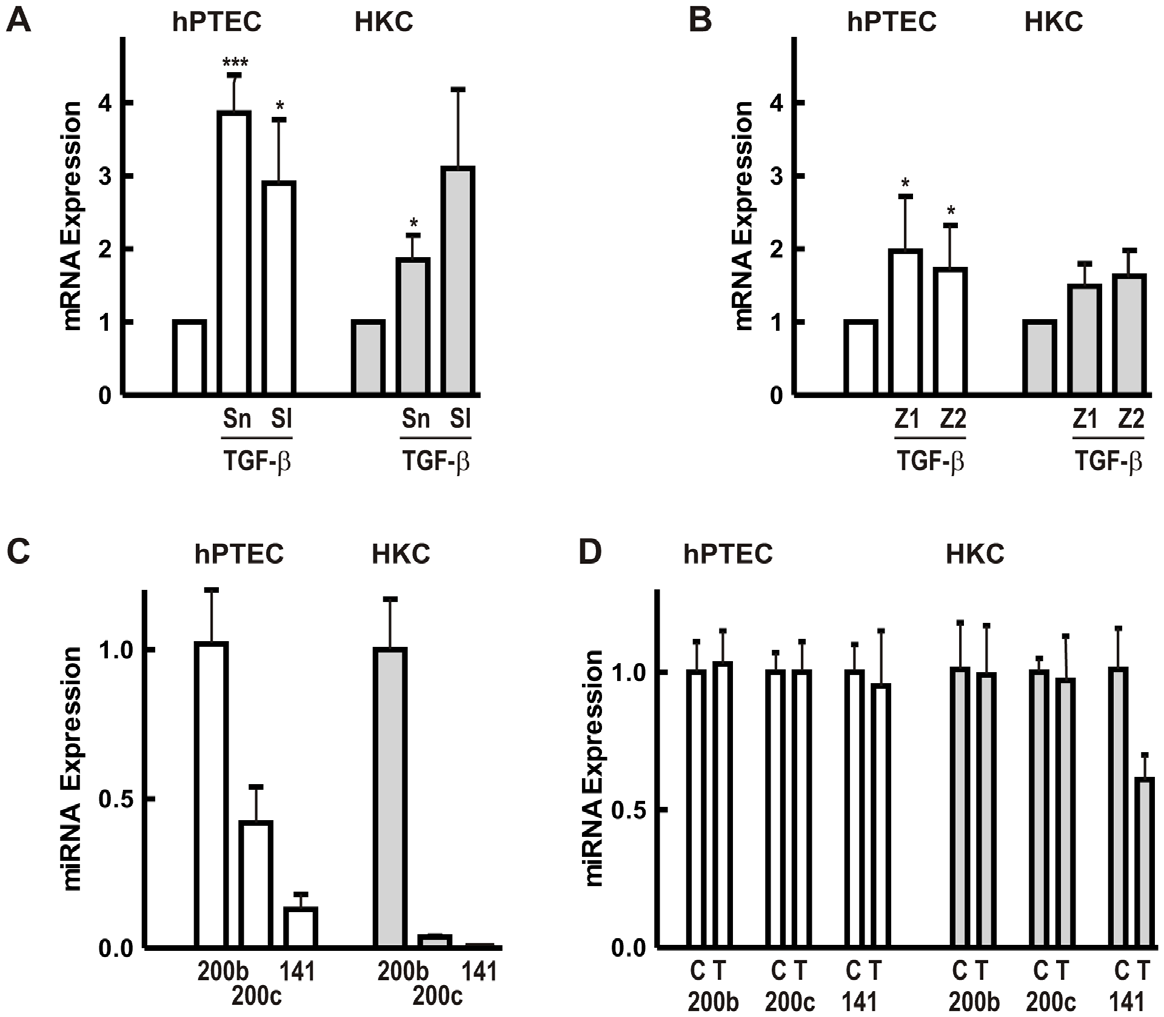
Regulation of transcription factors and miRNAs potentially involved in E-cadherin regulation. (A) hPTECs or HKC-8 cells were incubated with TGF-β (2 ng/ml) for 6 h. Snail (Sn) and Slug (Sl) mRNA expression was quantified by RT-PCR. Expression of Snail or Slug mRNA was set to 1 in each experiment. Data are means +/− SD of 4 preparations. ***p<0.001, *p<0.05, one way ANOVA with Dunnett’s post hoc test. (B): Cells were treated as in A. ZEB1 (Z1) and ZEB2 (Z2) was detected by RT-PCR. Data are means +/− SD of 6 different preparations of primary cells and 3 experiments with HKC-8 cells. *p<0.05, calculated as in 4A. (C) miRNA levels of miR200b, miR200c and miR141 were detected in 3 different preparations of hPTECs and compared to HKC-8 cells. In each set of experiments, expression of miR200b was set to 1. Error bars reflect variability of duplicate determinations. Error bars of miR200c and miR141 in HKC cells were smaller than the line thickness of the graph. (D) Cells were treated with TGF-β for 24 and 72 h (hPTECs) and 24 h (HKC-8). miRNA expression was determined by RT-PCR. Data are means +/− SD of 3 different preparations of primary cells and 2 experiments with HKC-8 cells analyzed in duplicate. Means of control cells (C) were set to 1 for each miRNA; T: TGF-β-treated cells.

Downregulation of E-cadherin is mediated by transcription factors of the Snail – Slug family [33]. In our study, Snail mRNA levels were comparable in hPTECs and HKC-8 cells. Slug was less abundant, especially in HKC-8 cells (ratio Snail to Slug mRNA 5∶1 in hPTECs and 50∶1 in HKC-8 cells). Treatment with TGF-β increased Snail and Slug mRNA expression in both cell types (Fig. 4A). Increased levels were still detectable after prolonged incubation for 24 and 72 h (Fig. S2). Upregulation of Snail or Slug mRNA was thus not sufficient to down-regulate E-cadherin expression in hPTECs.

It has been suggested that the interplay between transcription factors ZEB1 and ZEB2 and miRNAs of the miR200 family plays an essential role in TGF-β-mediated regulation of E-cadherin [34]. In hPTECs mRNA levels of ZEB1 and-2 were comparable to Snail, whereas in HKC-8 cells, expression of ZEB-2 mRNA was about 4fold higher than the other two transcription factors (Fig. S3). Regulation of ZEB1 and-2 mRNA by TGF-β was marginal in both cell types (Fig. 4B, 6 h). A transient upregulation was detectable after 6 h, but was no longer observed after 24 or 72 h.

**Figure 5:**
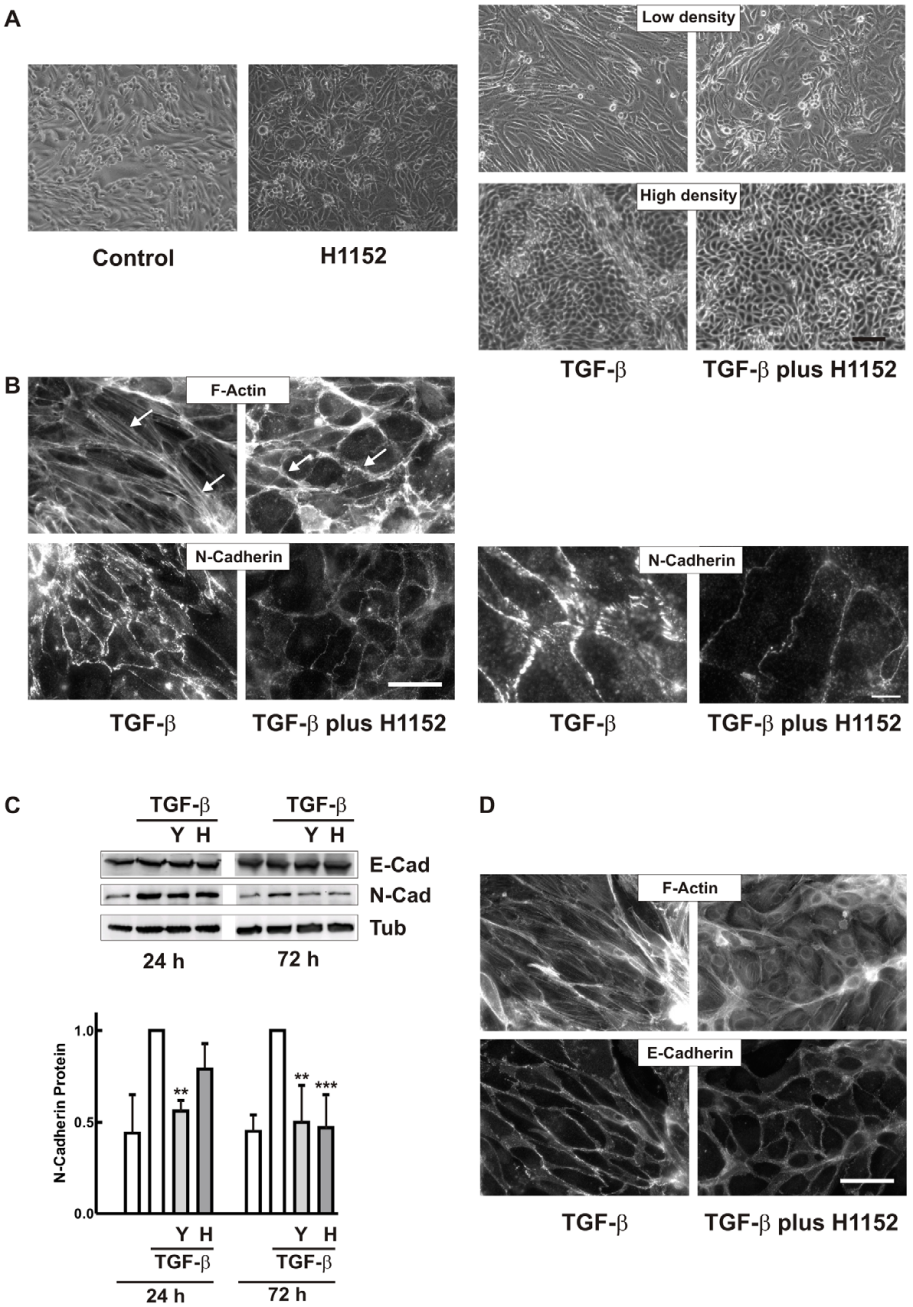
Inhibition of Rho kinases interferes with TGF-β-induced morphological alterations. (A) hPTECs cultured as control cells or were treated with H1152 (0.75 µM), TGF-β (2 ng/ml) or TGF-β plus H1152 as indicated for 72 h. Phase contrast images of cells seeded at low or high density are depicted. Scale bar: 100 µm: (B) N-cadherin was detected by immunocytochemistry in cells treated with TGF-β or TGF-β plus H1152 for 72 h. F-actin was visualized by rhodamine-phalloidin staining. Arrow heads indicate stress fibers and cortical F-actin, respectively. Scale bar: 50 µm. Right hand panels show N-cadherin structures at higher magnification. Scale bar: 10 µM. (C) hPTECs were treated with TGF-β or TGF-β plus 10 µM Y27632 (Y) or 0.75 µM H1152 (H) for 24 h and 72 h. E-cadherin and N-cadherin protein expression was detected by Western blotting. Expression of N-cadherin (means +/− SD of 3–4 independent preparations) was summarized with N-cadherin expression in TGF-β-treated cells set to 1 in each experiment. ***p<0.001, **p<0.01, one way ANOVA with Dunnett’s post hoc test. (D) E-cadherin and F-actin were detected in cells treated as in B.

Moreover, miRNAs of the miR200 family have not been analyzed in human tubular cells and therefore, we chose three members of the miR200 family to assess their abundance, namely 200b, 200c and 141. miR200b showed the highest miRNA expression with a comparable expression in hPTECs and HKC-8 cells (Fig. 4C). Compared to miR200b, which was used as reference in Fig. 5C, miR200c and miR141 were expressed less abundantly with a considerably higher expression in hPTECs compared to HKC-8 cells.

In this study, HKC-8 cells and hPTECs were treated with TGF-β up to 72 h, but no significant change in the expression of miR200b or miR200c was detectable (Fig. 4D). miR141 was downregulated in HKC-8 cells but not in hPTECs.

### Rho-kinase Inhibitors Counteract TGF-β-induced Structural Alterations

The strong morphological alterations observed in proximal tubular cells or subconfluent distal cells upon treatment with TGF-β prompted us to modulate actin-mediated contractility. As pharmacological tool we used non-selective inhibitors of Rho-kinases, main downstream mediators of RhoA and RhoC and regulators of F-actin structures. Phase contrast images showed marked effects of the Rho-kinase inhibitor H1152: after 72 h, control cells appeared more cobble-stone-like in the presence of the inhibitor (Fig. 5A). TGF-β-induced elongation or structure formation of hPTECs was strongly reduced (Fig. 5A). Comparable results were obtained with the chemically distinct Rho-kinase inhibitor Y27632 ( Fig. S4).

N-cadherin positive proximal hPTECs maintained more regular adherent structures in the presence of Rho-kinase inhibitors (Fig. 5B) TGF-β-induced stress fiber formation was prevented, whereas the cortical actin remained intact (arrow heads in Fig. 5B). Interdigitation of N-cadherin at cell-cell contacts was reduced to a fine line (Fig. 5B, right hand panels with higher magnification). In line with the weaker immunofluorescence, Rho-kinase inhibitors reduced TGF-β-mediated upregulation of N-cadherin protein expression as shown by Western blot analysis (Fig. 5C).

As outlined above, only subconfluent distal tubular cells were morphologically altered by TGF-β and only these cells were affected by Rho-kinase inhibitors. Co-incubation with these inhibitors reduced TGF-β−induced stress fibers and the cells did not elongate upon TGF-β treatment (Fig. 5D). E-cadherin protein expression was not affected by Rho-kinase inhibitors as also confirmed by Western blot analyses (blot in Fig. 5C).

**Figure 6:**
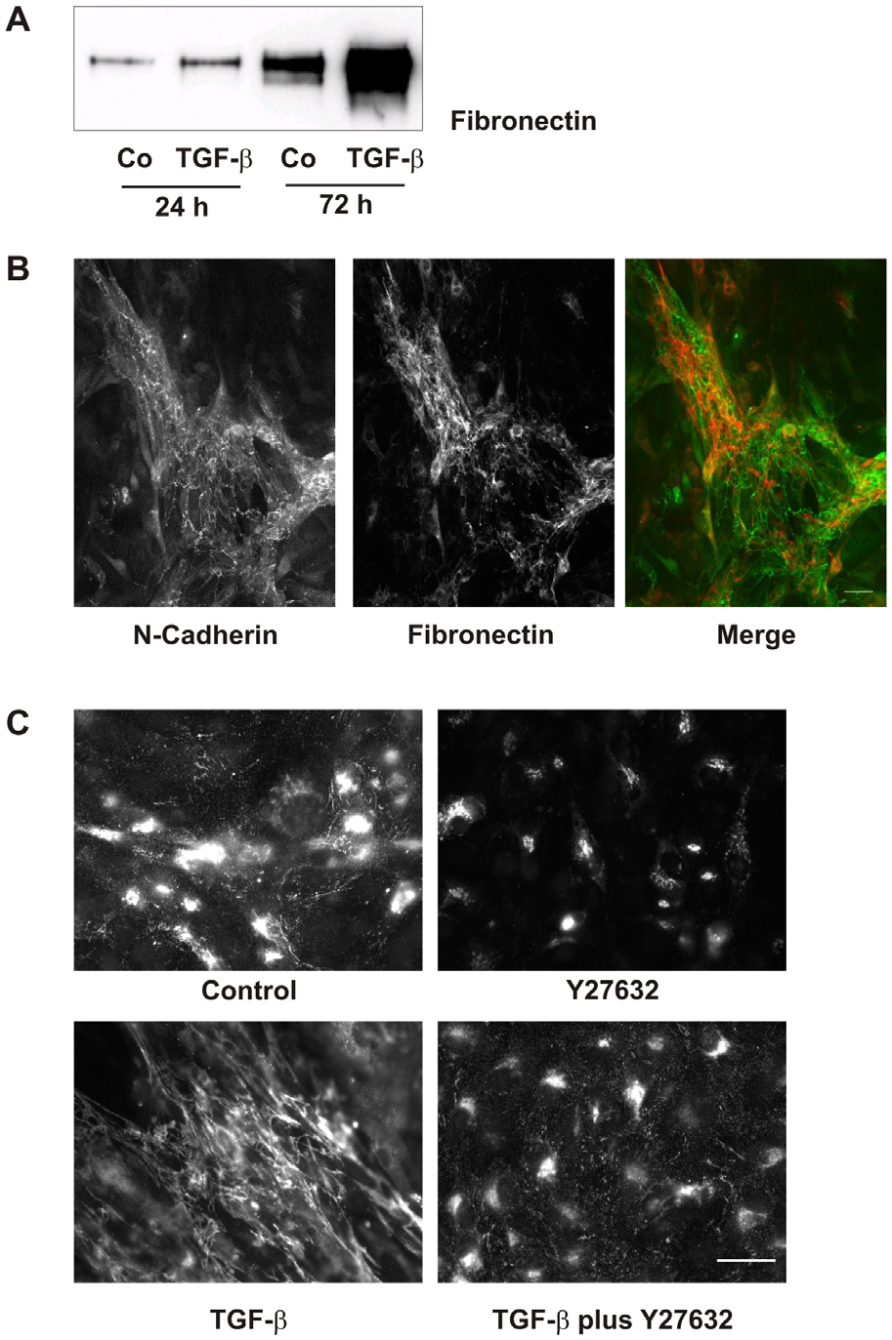
Formation of the fibronectin network is dependent on Rho-kinase activity. (A) hPTECs were incubated with TGB-β (2 ng/ml) for 24 and 72 h. Supernatants were precipitated and fibronectin was detected by Western blotting. (B) N-cadherin and fibronectin were visualized by immunocytochemistry in cells treated with TGF-β for 72 h. (C) The fibronectin network was detected in cells treated with TGF-β and the Rho-kinase inhibitor Y27632 (10 µM) for 72 h.

Furthermore, inhibition of Rho-kinases profoundly counteracted the effect of TGF-β on cell-matrix interactions. As an example we analyzed fibronectin secretion. Upon stimulation with TGF-β, fibronectin was secreted into the cell culture supernatant detectable by Western blotting (Fig. 6A). Furthermore, in the presence of TGF-β, fibronectin formed a network of fibers most pronounced in association with proximal tubular cells (Fig. 6B). These fibrous structures were not formed in the presence of Rho-kinase inhibitors (Fig. 6C).

### Rho-kinase Inhibitors Stabilize Epithelial Structures in Polarized Epithelia Cells

**Figure 7:**
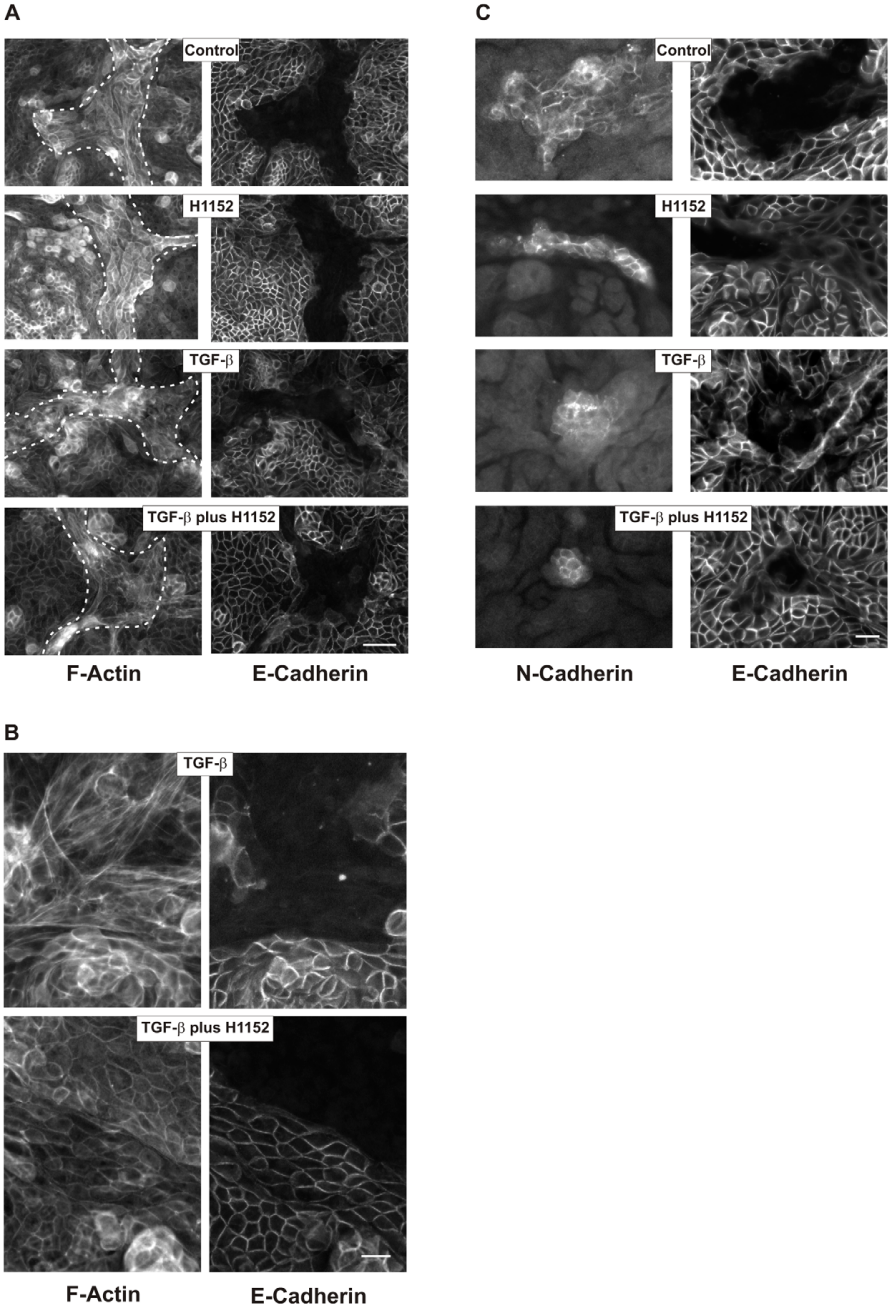
Structural alterations in polarized hPTECs. (A) hPTECs were incubated in permeable filter inserts for 8 days to achieve polarization. They were further incubated with TGF-β (2 ng/ml) and H1152 (0.75 µM) for 7 days. Cells were stained for F-actin and E-cadherin. N-cadherin proximal tubular cells are visualized by F-actin only and are marked by the dotted lines (left panels). Scale bar: 50 µm. (B) Higher magnification of F-actin fibers in polarized cells treated with TGF-β or with TGF-β plus H1152 as described in 7A. Scale bar: 20 µm. (C) Cells were treated as in 7A. Proximal and distal cells were detected by antibodies directed against N-and E-cadherin, respectively. Scale bar: 50 µm.

Next we investigated whether Rho-kinase inhibitors also affected polarized epithelial cells. Both, proximal and distal polarized hPTECs express primarily cortical F-actin fibers (Fig. 7).

Upon treatment with TGF-β F-actin structures became more irregular, most notably in proximal hPTECs (Fig. 7A/B). Incubation with Rho-kinase inhibitors stabilized the epithelial phenotype and prevented TGF-β-induced morphological changes most obviously in proximal cells (Fig. 7C). Cortical F-actin fibers remained preserved in cells co-incubated with TGF-β and H1152 (Fig. 7B).

### Inhibition of Rho-kinase Isoforms Distinctly Alters F-actin Cytoskeleton

**Figure 8:**
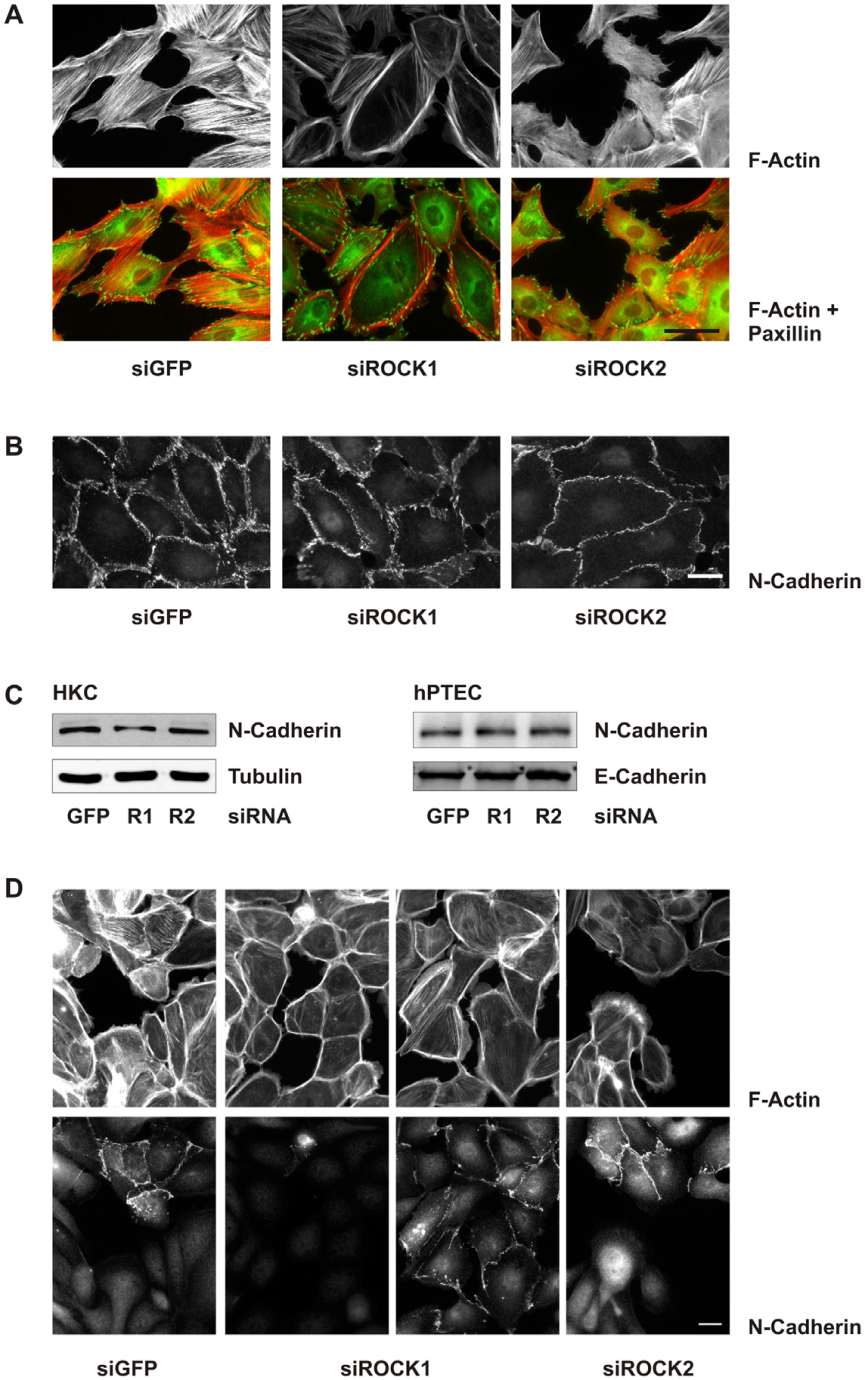
Isoform-specific changes in F-actin fiber formation by ROCK1 and ROCK2 siRNA. (A) HKC-8 cells were transfected with siRNAs as indicated. After 48 h the cells were stimulated with LPA (10 µM) for 2 h. Cells were stained for F-actin (red) and paxillin (green). Scale bar: 50 µm. (B) HKC-8 cells were treated as in A. N-cadherin was visualized by immunocytochemistry. Scale bar: 20 µm. (C) HKC-8 cells or hPTEC were transfected with siRNAs against ROCK1 (R1), ROCK2 (R2) or GFP as indicated. After 72 h, expression of N-cadherin, E-cadherin, and tubulin was detected by Western blotting. (D) hPTECs were treated as in A. Cells were stained for F-actin and for N-cadherin to distinguish between proximal and distal cells.

H1152 and Y27632 are non-selective inhibitors of both Rho-kinase isoforms, ROCK1 and ROCK2. To analyze isoform-specific effects we used a siRNA approach which was established in HKC-8 cells. Changes in F-actin structures were most obvious in subconfluent cells treated with lysophosphatidic acid to activate Rho-Rho-kinase signaling (Fig. 8A). Downregulation of ROCK1 markedly reduced cell spanning F-actin fibers whereas cortical fibers were enhanced. By contrast, downregulation of ROCK2 induced a network of shorter intracellular fibers and destabilized the cortical F-actin leading to the formation of invaginations in peripheral cells. These alterations in F-actin fibers were reflected at the level of focal adhesions visualized by staining of paxillin, which were organized at the cell periphery in siROCK1 cells and marked the irregular structures in siROCK2 cells. Interdigitating N-cadherin structures were reduced by siROCK2, reminiscent of the effects of non-selective inhibitors (Fig. 8B), although protein expression of N-cadherin was not reduced in siROCK2-treated cells (Fig. 8C). Comparable data were obtained with hPTECs (Fig. 8C, right panel).

**Figure 9:**
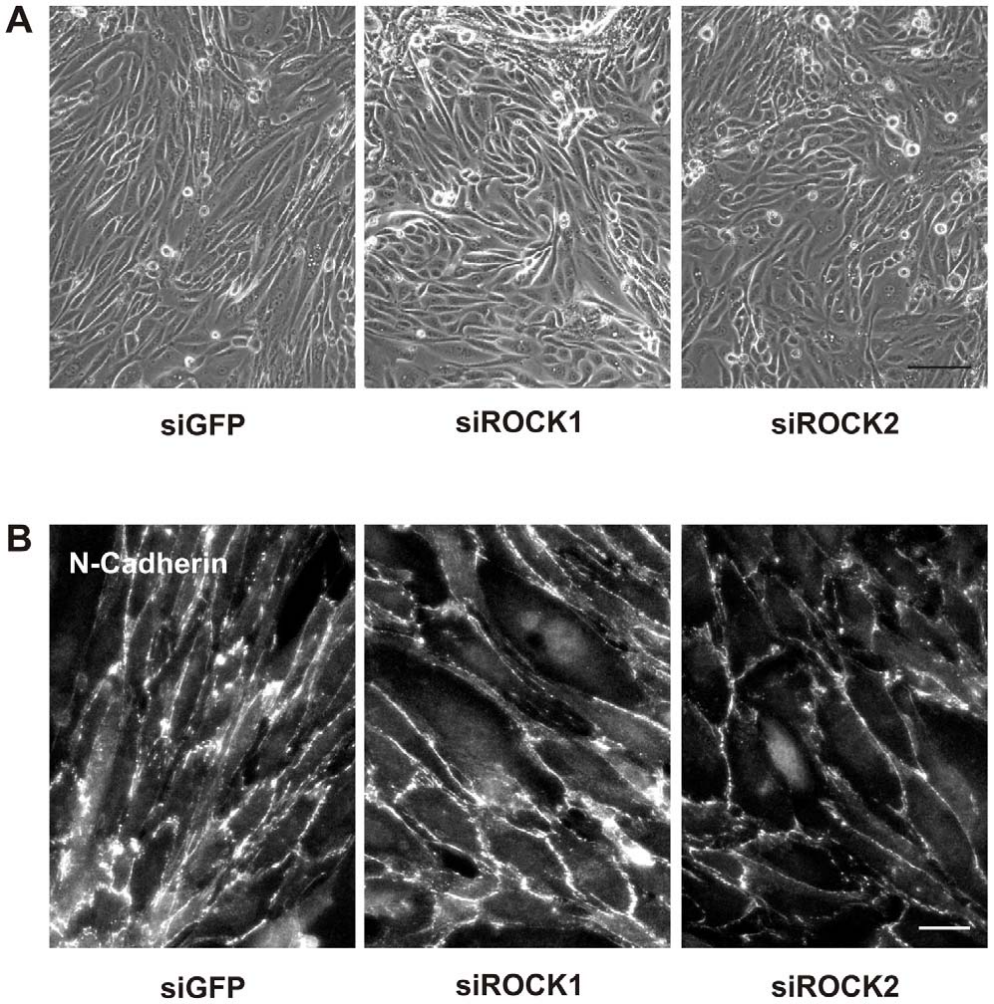
Isoform-specific morphological alterations in proximal tubular epithelial cells. (A) hPTECs were transfected with siRNAs as indicated and then treated with TGF-β for 72 h. Phase contrast images are representative for 5 different preparations. Scale bar: 100 µm. (B) hPTECs were treated as in A: N-Cadherin was visualized by immunocytochemistry. Scale bar: 20 µm.

F-actin structures were more variable in hPTECs. siROCK1-mediated loss of cell spanning F-actin fibers and stabilization of cortical F-actin was observed in proximal as well as distal cells, whereas dissolution of cortical F-actin by siROCK2 was most obvious in distal hPTECs (Fig. 8D). Downregulation of either isoform reduced TGF-β-mediated elongation of hPTECs observed after 72 h (Fig. 9A). Proximal hPTECs remained spread and less aligned compared to siGFP-transfected cells (Fig. 9B). As expected, effects were more moderate compared to non-selective inhibition of Rho-kinases.

## Discussion

Polarized and non-polarized hPTECs of proximal and distal renal tubular origin showed pronounced differences in morphological plasticity. Most markedly, proximal cells showed a high flexibility and readily underwent morphological alterations, whereas distal cells remained stable and did not downregulate E-cadherin as expected based on data obtained with tumor cells or cell lines. Analysis of molecular mechanisms showed a role for Rho-kinases in hPTECs plasticity, and stable expression of ZEB transcription factors and miRNAs of the miR200 family as potential mediators of E-cadherin stability in distal cells.

Primary cultures of hPTECs consist of cells of different origin best distinguishable at passage 1 or 2 after isolation when cells formed large clusters of either proximal or distal cells. Formation of confluent monolayers proved to be a critical parameter in the analyses. Distal hPTECs reacted strongly to TGF-β when subconfluent, but retained their phenotype when cell-cell contacts were stabilized by E-cadherin. In line with these observations, Elberg et al. [35] observed mesenchymal alterations in serum-cultured subconfluent human tubular cells, the renal origin of which was not further characterized. Confluent cells may approximate cells in intact tubules whereas subconfluent cells behave more like cells during tubular injury. In line with our studies, Koester et al. did not detect signs of mesenchymal transition in an in vivo model with inducible TGF-β over-expression in renal tubules, where TGF-β was the driving force acting on intact tubules [36].

Most strikingly, distal tubular cells did not downregulate E-cadherin expression upon exposure to TGF-β. This was not due to a loss of reactivity to TGF-β as shown by translocation of Smads to the nucleus. By contrast and in line with reports in the literature, E-cadherin expression was rapidly reduced in HKC-8 cells, which are of proximal tubular origin and express both, N-and E-cadherin. Regulation of E-cadherin expression by TGF-β is orchestrated by interacting transcription factors including Snail, Slug, ZEB1 and ZEB2 [33]. In HKC-8 cells and even stronger in hPTECs, we observed a rapid upregulation of Snail and Slug mRNA, which correlated with E-cadherin downregulation in HKC-8 cells but not in our primary cells. Interaction with other signaling pathways seems to be essential for downregulation of E-cadherin by Snail/Slug transcription factors as analyzed in MDCK cells [37], [38], [39]. Complex formation between MKL1 and Smad3 to upregulate Slug [37] or cooperation between Snail and LEF-1 transcriptions factors [38] demonstrated the complexity of E-cadherin regulation even within one cell line. Furthermore, posttranscriptional regulation of the transcription factors may contribute to the differences between HKC-8 cells and hPTECs.

Regulation of renal development, homeostasis and pathology by miRNAs is a newly developing field of research [40], [41], [42]. Recently, several groups showed a mutual negative feedback loop between ZEB1/2 and miRNAs of the miR200 family regulating cellular plasticity in cancer cells but also in renal tubular cell lines [34]. Expression of ZEB1 and ZEB2 was upregulated concomitant with downregulation of miR200b and miR200c in TGF-β-treated cell lines, MDCK cells and normal rat kidney epithelial (NRKE) cells [43], [44], [45]. Prolonged autocrine signaling of TGF-β was necessary to drive sustained ZEB upregulation and miR200 silencing for maintenance of the mesenchymal state. Neither HKC-8 cells nor hPTECs showed prolonged upregulation of ZEB1 or ZEB2 upon treatment with TGF-β. Correspondingly, there was no significant regulation of miR200b, 200c or 141 in hPTECs. In HKC-8 cells, miR141 was reduced by 25%. Nevertheless, the biological significance remains to be determined as miR141 had by far the lowest expression level. Given the selective regulation of miR141, a role of further members of the miR200 family, e.g. miR200a or miR429 cannot be excluded. Interestingly, the concentration of miRNAs was much higher in primary cells compared to HKC-8 cells, (miR200b: 10fold; miR141∶100fold). In tumor cells, high levels of miRNA200 have been associated with the epithelial phenotype compared to low levels being associated with mesenchymal phenotypes. Our data are thus in line with the hypothesis that high levels of miRNAs of the miR200 family and the missing regulation by TGF-β contribute to the stability of E-cadherin expression in distal tubular cells.

A much higher morphological plasticity was observed in tubular epithelial cells of proximal origin. These cells lost contact with the matrix and formed three dimensional structures, even more so when stimulated with TGF-β. This phenomenon was observed also in polarized cells, where proximal cells appeared to be mechanically stressed by surrounding distal cells. Proximal tubular cells are the only epithelial cells of the adult human organism which express N-cadherin under normal circumstances and not as a sign of pathological mesenchymal transition [3], [4], [46]. Homophilic N-cadherin binding is less strong than homophilic E-cadherin binding [47]. Both cadherins also differ in their intracellular binding partners which may affect cell motility [48]. Therefore, though certainly not exclusively, the difference in cell-cell adhesion molecules may contribute to the higher plasticity observed in tubular cells of proximal origin.

Morphological changes due to TGFbeta signaling have been attributed to direct or indirect activation of Rho proteins [49], [50] and inhibition of Rho kinases was antifibrotic in several models of renal diseases [42], [51], [52], [53]. We have shown earlier that Rho-kinase inhibitors increased migration velocity of tubular epithelial cells in wound healing assays, and may thus contribute to increased epithelia repair after injury [54]. In this study we provide evidence that inhibition of Rho-kinases also interferes with TGF-β-induced mesenchymal alterations, most obviously in proximal tubular epithelial cells but also subconfluent distal cells, which are not stabilized by a well structured environment.

Most interestingly, inhibition of Rho-kinases stabilized F-actin fibers in polarized cells at the cell-cell boundaries, the organization of which is primarily attributed to mDIA kinases, which are also activated by RhoA [55], [56]. This shift in downstream mediators of RhoA or RhoC may be essential for the protective role of Rho-kinase inhibitors in addition to alterations in gene expression.

Inhibition of Rho-kinases also affected the architecture of the extracellular matrix as shown by the breakdown of fibronectin fibers. Further studies are necessary to analyze the mutual interaction between structural alteration of the cells and network formation of the extracellular matrix in the context of mesenchymal alterations.

The effects of Rho-kinase inhibitors go beyond anti-fibrotic actions. Most notably, by affecting vascular smooth muscle cells, non-selective Rho-kinase inhibitors cause vasodilatation [57], [58] and are presently tested for their antihypertensive potential also in humans. Therefore, we asked the question whether inhibition of one of the Rho-kinase isoforms might be a relevant pharmacological target to modulate hPTECs mesenchymal alterations. Selective knock-down of either ROCK1 or ROCK2 differentially affected cytoskeletal organization. However, both isoforms played a role in the complex alterations detected after 72 h of TGF-β treatment, which represent a composite of multiple changes in signaling mediators and enzyme activities occurring in a coordinated timely and spatial manner. Although the details of ROCK1-and ROCK2-mediated cellular effects in hPTECs need further evaluation our data already suggest that targeting of Rho-kinase isoforms may be promising to modulate tubular plasticity sparing the profound vascular effects of non-selective inhibitors.

In conclusion, cultures of freshly isolated hPTECs reflect functional differences between cells of different tubular origin expressing N-cadherin and E-cadherin, respectively, as major cell-cell adhesion molecules. Most notably, characteristics of mesenchymal alterations and responsiveness to TGF-β signaling distinguish primary cells from cell lines. Therefore, cultures of hPTECs are a promising system to further delineate the complex molecular alterations of epithelial cells observed in renal injury and regeneration.

## Materials and Methods

DMEM/Ham’s F12 medium was purchased from Biochrom AG (Berlin, Germany), DMEM medium and Hanks BSS from PAA Laboratories (Coelbe, Germany), insulin-transferrin-selenium supplement from Gibco (Karlsruhe, Germany), fetal calf serum (FCS) from PAN Biotech (Aidenbach, Germany), triiodothyronine from Fluka (Buchs, Switzerland), hydrocortisone from Sigma (Munich, Germany), epidermal growth factor from PeproTech (Hamburg, Germany), TGF-β1 from tebu-bio (Offenbach, Germany), lysophosphatidic acid (LPA) (Sigma-Aldrich, Munich, Germany); Y27632, (+)-(*R*)-*trans*-4-(1-aminoethyl)-*N*-(4-pyridyl) cyclohexanecarboxamide dihydrochloride. H1152 (*S*)-(+)-2-methyl-1-[(4-methyl-5-isoquinolinyl)sulfonyl]-homopiperazine [59] (ALX-270-423-M001, Alexis Biochemicals, Grünberg, Germany),

### Cell Culture

Human primary tubular epithelial cells (hPTECs) were isolated from renal cortical tissues collected from healthy parts of tumor-nephrectomies essentially as described previously [3]. Isolation of human cells from healthy parts of tumor nephrectomies was approved by the local ethics committee (Ethik-Kommission der Medizinischen Fakultät der Friedrich-Alexander Universität Erlangen-Nürnberg). The named institutional ethics committee specifically approved the use of kidney material (Reference number 3755). We obtained written informed consent from all participants involved in this study. In brief, after transport in Hank’s BSS, cortex tissue was cut into 1 mm^3^ pieces and digested with collagenase type II (Gibco, Karlsruhe, Germany) and DNase I grade II (Roche Diagnostics, Mannheim, Germany) for 60 min. Next, cell suspension was sieved through 100 µm and 70 µm meshes. After a washing step with HBSS, cells were seeded in epithelial cell selective medium (DMEM/Ham’s F12 medium containing 2 mM L-glutamine, 100 U/ml penicillin, 100 µg/ml streptomycin, insulin-transferrin-selenium supplement, 10 ng/ml epidermal growth factor, 36 ng/ml hydrocortisone and 4 pg/ml triiodothyronine) in the presence of 0.5% FCS. After 1–2 days, medium was replaced by FCS-free medium. Cells were subcultured by application of trypsin. For experiments, hPTECs were seeded in medium containing 2.5% FCS to facilitate cell attachment, and medium was replaced after 24 h to FCS-free epithelial cell selective medium. Bright field pictures were recorded by Olympus CK40 microscope (Olympus, Hamburg, Germany) using Leica DC Viewer software (Leica, Herbrugg, Switzerland).

About five days after isolation at passage one, cells were routinely analyzed for the content of proximal and distal cell by staining for N-and E-cadherin, respectively. The ratio of distal and proximal cells varied with most preparation containing a higher percentage of distal cells (about 70%).

Polarized tubular epithelial cells were obtained by culturing primary epithelial cells for 8 days on permeable transwell inserts (Millicell, Millipore, Schwalbach, Germany).

HKC-8 cells were cultured as described previously [60].

### Western Blot Analysis

Cells were lyzed in buffer containing 50 mM HEPES pH 7.4, 150 mM NaCl, 1% Triton X-100, 1 mM EDTA, 10% glycerol, 2 mM sodium vanadate and protease inhibitors complete EDTA-free (Roche Diagnostics, Mannheim, Germany). Western blot analyses were performed essentially as described before [60] using the antibodies listed as Table S1A. To ensure equal loading and blotting, blots were redetected with an antibody directed against tubulin or vinculin. The immunoreactive bands were quantified using the luminescent image analyzer (LAS-1000 Image Analyzer, Fujifilm, Berlin, Germany) and AIDA 4.15 image analyzer software (Raytest, Berlin, Germany).

### siRNA Transfection

To down-regulate ROCK1 and ROCK-2 epithelial cells were transfected 3 h after seeding using HiPerFect (QIAGEN GmbH, Hilden, Germany) according to the manufacturer’s instructions. siRNA directed against GFP was used as control. Experiments were performed 48 h after transfection.

Different specific siRNAs and siRNA against GFP (for details see Table S1B) were used to rule out non-specific effects, off target effects or alterations due to the transfection procedure. ROCK1 protein was consistently down-regulated by about 90% whereas downregulation of ROCK2 reached 75% (Figure S5). Silencing of one isoform did not induce compensatory upregulation of the other isoform as observed in other cell systems [30].

### RNA-Isolation and Real-time RT-PCR

Total RNA was prepared from cultured epithelial cells using TriFast reagent from Peqlab (Erlangen, Germany). 100 ng RNA were reverse transcribed with TaqMan reverse transcription reagents (Applied Biosystems, Foster City, CA, USA) according to the manufacturer’s instructions. cDNA was amplified using Power SYBR MM reaction buffer (Applied Biosystems). The PCR reactions were carried out using the ABI PRISM 7000 Sequence Detection System (Applied Biosystems). Relative RNA was calculated using the delta-delta ct method. Primers used for RT-PCR are listed in Table S1C.

miRNAs were isolated and quantified using miRNA miScript II Reverse Transcription Kit (Qiagen, Hilden, Germany). miScript primer assays (Qiagen, Hilden, Germany) were used to detect miRNA expression following the manufacturer’s instructions Hs_miR-200c_1, Hs_miR-200b_3, Hs_miR-141_1 were used to detect members of the miR200 family and. Hs-SNORD44-1 was used as control.

### Immunocytochemistry

Cells were fixed with paraformaldehyde (3.5% in PBS) for 10 min and afterwards permeabilized by 0.5% Triton X-100 in PBS for 10 min. After washing three times with PBS, cells were blocked in 1% BSA in PBS for 1 h at room temperature and washed once. Antibodies used for Immunocytochemistry are listed as Table S1D. After mounting, slides were viewed using a Nikon Eclipse 80i fluorescent microscope and digital images recorded by Visitron Systems 7.4 Slider camera (Diagnostic Instruments, Sterling Heights, MI, USA) using Spot Advanced software (Diagnostic Instruments). Three dimensional images were evaluated by epifluorescence microscopy including ApoTome technique (Zeiss, Göttingen, Germany).

### Statistical Analysis

To compare multiple conditions, statistical significance was calculated by one-way ANOVA with Dunnett’s post hoc test, or one sample t-test using GraphPad software. A value of P<0.05 was considered to indicate significance.

## Supporting Information

Figure S1 **Characterization of polarized hPTECs.** Polarized hPTECs were stained for E-cadherin and peanut lectin (PNA) or N-cadherin and aminopeptidase N (CD13) as indicated. Scale bar: 20 µm.
(PDF)

Figure S2 **Long-term regulation of Snail and Slug mRNA in hPTECs and HKC cells by TGF-β.** hPTECs (S2A) or HKC-8 cells (S2B) were incubated with TGF-β (2 ng/ml) for 24 or 72 h. Snail and Slug mRNA expression was quantified by RT-PCR. Data are means +/− half range of 2 preparations (hPTECs) or means +/− SD of 3 experiments (HKC). In each experiment expression of control cells at the respective time point was set to 1. Error bars of control cells reflect errors of duplicate PCR analyses. ***p<0.001, **p<0.01 one way ANOVA with Dunnett’s Multiple comparison test.
(PDF)

Figure S3 **Semi quantitative comparison of Snail, ZEB1 and ZEB2 mRNA expression.** S3A: mRNA of 3 experiments performed with 2 preparations of hPTECs was analyzed on one plate for Snail, ZEB1, ZEB2 and 18S mRNA expression in duplicate. Expression of Snail was set to 1 in each experiment. The error bar of Snail reflects the variability of the duplicates. S3B: Snail, ZEB1 (Z1) and ZEB2 (Z2) mRNA expression was determined in HKC-8 cells. A typical example is shown in the left part, samples analyzed in duplicate. Expression of ZEB1 and ZEB2 was compared in 5 experiments (right part). Expression of ZEB1 mRNA was set to 1 in each experiment. ***p<0.001, one sample t-test.
(PDF)

Figure S4 **Inhibition of Rho kinases by Y27632 prevents TGF-β-induced morphological alterations.** hPTECs were treated with TGF-β (2 ng/ml) and/or the Rho kinase inhibitor Y27632 (10 µM) for 72 h as indicated. Structural alterations are shown by phase contrast images. Scale bar: 200 µm.
(PDF)

Figure S5 **Regulation of ROCK1 and ROCK2 by siRNA in hPTECs and HKC-8 cells.** hPTECs and HKC-8 cells were transfected with siRNAs directed against ROCK1 (R1), ROCK2 (R2) or GFP. 48 h after transfection, ROCK1 and ROCK2 were detected in cellular homogenates by Western blotting. Expression of either isoform in GFP-treated cells was set to 1. Data are means +/− SD of 4 experiments with 2 different siRNAs each. Expression of ROCK1 (R1) or ROCK2 (R2) in GFP-treated cells was set to 1 in each experiment. Compared to GFP-treated cells, specific downregulation of the respective ROCK mRNA was significant (p<0.01 in hPTECs and p<0.001 in HKC8 cells, one sample t-test).
(PDF)

Table S1 S1A: Antibodies used for Western blotting. S1B: siRNAs. S1C: Primers used for RT-PCR. S1D: Antibodies used for immunocytochemistry
(PDF)
