# Supplementary figures and images for: Distinct Mesenchymal Alterations in N-Cadherin and E-Cadherin Positive Primary Renal Epithelial Cells
Source: PLoS One. 2012 Aug 17;7(8):e43584. doi: 10.1371/journal.pone.0043584 (PMC3422254; doi:10.1371/journal.pone.0043584)

## Supplementary Figure S1

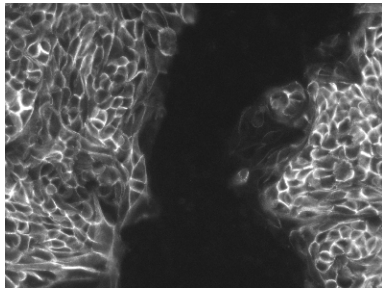

**E-Cadherin**

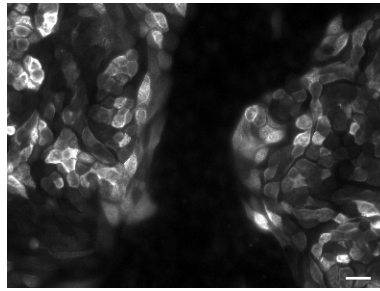

**Peanut Agglutinin**

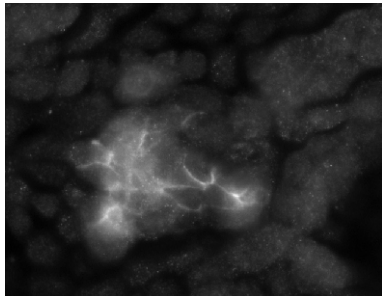

**N-Cadherin**

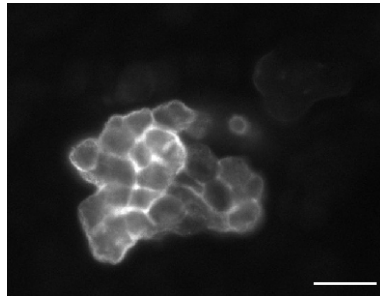

**Aminopectidase N**

Supplement: Figure S1 — Characterization of polarized hPTECs. Polarized hPTECs were stained for E-cadherin and peanut lectin (PNA) or N-cadherin and aminopeptidase N (CD13) as indicated. Scale bar: 20 µm. (PDF) [file pone.0043584.s001.pdf]

## Supplementary Figure S2

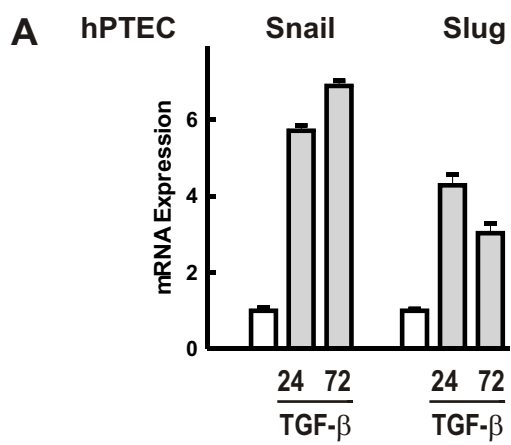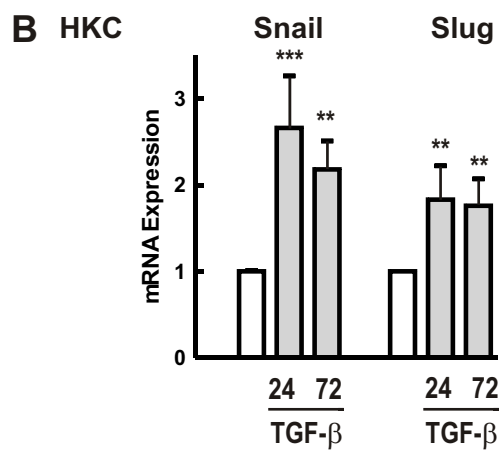

Supplement: Figure S2 — Long-term regulation of Snail and Slug mRNA in hPTECs and HKC cells by TGF-β. hPTECs (S2A) or HKC-8 cells (S2B) were incubated with TGF-β (2 ng/ml) for 24 or 72 h. Snail and Slug mRNA expression was quantified by RT-PCR. Data are means +/− half range of 2 preparations (hPTECs) or means +/− SD of 3 experiments (HKC). In each experiment expression of control cells at the respective time point was set to 1. Error bars of control cells reflect errors of duplicate PCR analyses. ***p<0.001, **p<0.01 one way ANOVA with Dunnett’s Multiple comparison test. (PDF) [file pone.0043584.s002.pdf]

## Supplementary Figure S3

### A hPTEC

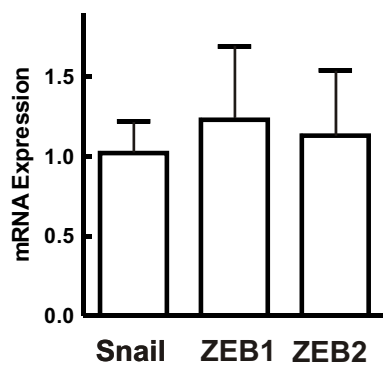

### B HKC

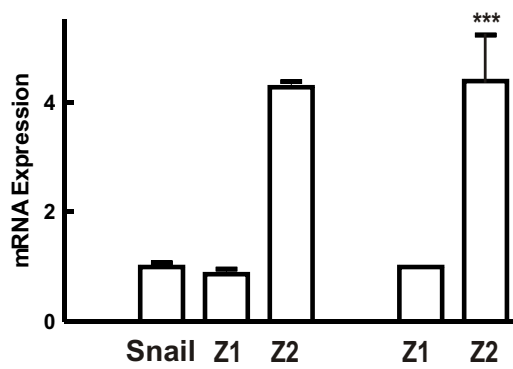

Supplement: Figure S3 — Semi quantitative comparison of Snail, ZEB1 and ZEB2 mRNA expression. S3A: mRNA of 3 experiments performed with 2 preparations of hPTECs was analyzed on one plate for Snail, ZEB1, ZEB2 and 18S mRNA expression in duplicate. Expression of Snail was set to 1 in each experiment. The error bar of Snail reflects the variability of the duplicates. S3B: Snail, ZEB1 (Z1) and ZEB2 (Z2) mRNA expression was determined in HKC-8 cells. A typical example is shown in the left part, samples analyzed in duplicate. Expression of ZEB1 and ZEB2 was compared in 5 experiments (right part). Expression of ZEB1 mRNA was set to 1 in each experiment. ***p<0.001, one sample t-test. (PDF) [file pone.0043584.s003.pdf]

## Supplementary Figure S4

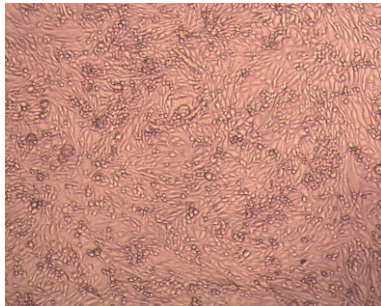

**Co**

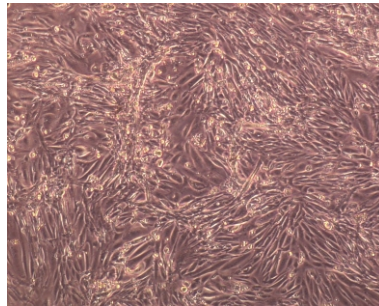

**TGF- $\beta$**

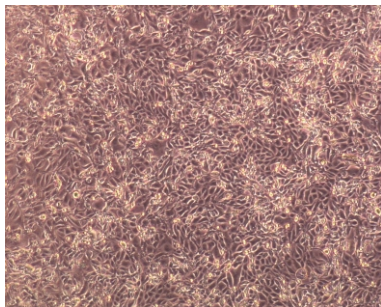

**Y27632**

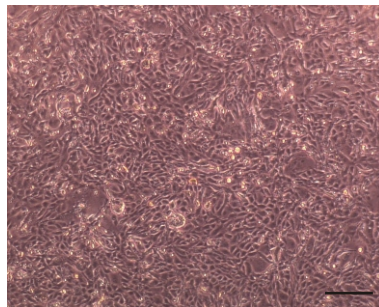

**TGF- $\beta$  + Y27632**

Supplement: Figure S4 — Inhibition of Rho kinases by Y27632 prevents TGF-β-induced morphological alterations. hPTECs were treated with TGF-β (2 ng/ml) and/or the Rho kinase inhibitor Y27632 (10 µM) for 72 h as indicated. Structural alterations are shown by phase contrast images. Scale bar: 200 µm. (PDF) [file pone.0043584.s004.pdf]

Supplementary Figure S5

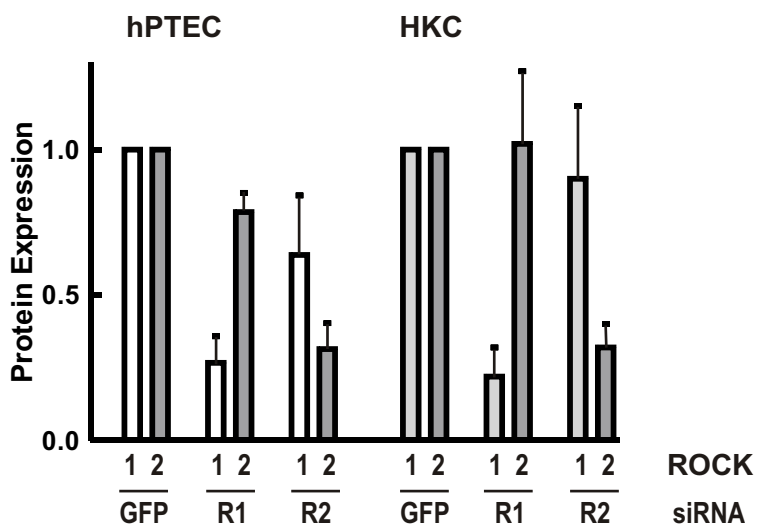

Supplement: Figure S5 — Regulation of ROCK1 and ROCK2 by siRNA in hPTECs and HKC-8 cells. hPTECs and HKC-8 cells were transfected with siRNAs directed against ROCK1 (R1), ROCK2 (R2) or GFP. 48 h after transfection, ROCK1 and ROCK2 were detected in cellular homogenates by Western blotting. Expression of either isoform in GFP-treated cells was set to 1. Data are means +/− SD of 4 experiments with 2 different siRNAs each. Expression of ROCK1 (R1) or ROCK2 (R2) in GFP-treated cells was set to 1 in each experiment. Compared to GFP-treated cells, specific downregulation of the respective ROCK mRNA was significant (p<0.01 in hPTECs and p<0.001 in HKC8 cells, one sample t-test). (PDF) [file pone.0043584.s005.pdf]
