# Supplementary material for: Distinct Mesenchymal Alterations in N-Cadherin and E-Cadherin Positive Primary Renal Epithelial Cells
Source: PLoS One. 2012 Aug 17;7(8):e43584. doi: 10.1371/journal.pone.0043584 (PMC3422254; doi:10.1371/journal.pone.0043584)
Supplement: Table S1 — S1A: Antibodies used for Western blotting. S1B: siRNAs. S1C: Primers used for RT-PCR. S1D: Antibodies used for immunocytochemistry (PDF) [file pone.0043584.s006.pdf]

Supplemental Table 1

Supplemental Table 1A: Antibodies used for Western blotting:

E-cadherin	rabbit	Cell Signaling
N-cadherin	rabbit	Santa Cruz
ROCK1	mouse	Santa Cruz
ROCK2	rabbit	Santa Cruz
Vinculin	rabbit	Santa Cruz
Fibronectin - HFN 7.1	mouse	DSHB
Tubulin	mouse	Sigma

Santa Cruz, Heidelberg, Germany:

Cell Signaling, Danvers, MA, USA

Sigma Sigma-Aldrich, Munich, Germany

DSHB: Developmental Studies Hybridoma Bank, The University of Iowa, Department of Biology, Iowa City, IA 52242.

HRP-conjugated secondary antibodies were from GE Healthcare Bio-Sciences, Uppsala, Sweden.

Supplemental Table 1B: siRNAs

ROCK1	Sense 5'-CCAGGAAGGUAUAUGCUAU-3'	Eurogentec
	esiRNA human ROCK1 (HU-03470-1)	Sigma
ROCK2	Sense 5'-GGAGAUUACCUUACGGAAA-3'	Eurogentec
	esiRNA human ROCK2 (HU-03013-1)	Sigma
GFP	Sense 5'-GGUGUGCUGUUUGGAGGUC-3'	Eurogentec

Eurogentec, Liege, Belgium

Sigma-Aldrich, Munich, Germany

Supplemental Table 1C: Primers used for RT-PCR

	Sense primer, 5' – 3'	antisense primer, 5' – 3'
E-Cadherin	CAT TGC CAC ATA CAC TCT CTT	TGC ATT CCC GTT GGA TGA C

	CTC TC	
N-Cadherin	CCC TGC TTC AGG CGT CTG TA	TGC TTG CAT AAT GCG ATT TCA CC
Snail	GGA AGC CTA ACT ACA GCG AGC T	GCT GGA AGG TAA ACT CTG GAT TAG A
Slug	AAG GAC ACA TTA GAA CTC ACA CGG	TGC AGT GAG GGC AAC AAA AAG
ZEB1	GAA AGA GAA GGG AAT GCT AAG AAC TG	CTT GCC CTT CCT TTC TGT CAT C
ZEB2	GCA CAA GAC TAC ATG TCA GGC C	CAC ACT GAT AGG GCT TCT CGC
18S	TTG ATT AAG TCC CTG CCC TTT GT	CGA TCC GAG GGC CTC ACT A

Supplemental Table 1D: Antibodies used for immunocytochemistry

E-cadherin	mouse	Abcam
N-cadherin	rabbit	Santa Cruz
Paxillin	mouse	Santa Cruz
Fibronectin - HFN 7.1	mouse	DSHB
Acetylated tubulin	mouse	Sigma
Rhodamine-phalloidin		Invitrogen
FITC-labeled CD13	mouse	Immunotools
FITC-labeled Peanut agglutinin		Sigma

Abcam, Cambridge, UK

Santa Cruz, Heidelberg, Germany

Sigma-Aldrich, Munich, Germany

Invitrogen, Karlsruhe, Germany

DSHB: Developmental Studies Hybridoma Bank, The University of Iowa, Department of Biology, Iowa City, IA 52242.

Immunotools, Friesoythe, Germany

Secondary Alexa Fluor 488- or -555-conjugated antibodies were from Invitrogen, Karlsruhe, Germany.
